# Construction and validation of a risk prediction model for postoperative urinary tract infection in intracranial hemorrhage patients

**DOI:** 10.3389/fmed.2025.1552191

**Published:** 2025-11-21

**Authors:** Ke Jiang, Yaqiang Li, Zhongbo Sun, Fu Chang, Yangyang Chen

**Affiliations:** 1 Department of Neurosurgery, First Affiliated Hospital of Anhui University of Science and Technology (First People’s Hospital of Huainan), Huainan, China; 2 Department of Neurology, People's Hospital of Lixin Country, Bozhou, China

**Keywords:** urinary tract infection, intracranial hemorrhage, nomogram, tumor necrosis factor-*α*, stroke

## Abstract

**Objectives:**

This study aimed to explore the risk factors for postoperative urinary tract infection (UTI) in patients with intracranial hemorrhage (ICH) and to establish and validate a nomogram that integrates predictive factors to estimate the likelihood of postoperative UTI following ICH.

**Methods:**

This study enrolled surgical patients with intracerebral hemorrhage (ICH) from January 2020 to November 2023, categorizing them into training and validation groups. Using multivariate logistic regression analysis, we identified significant predictors of postoperative UTI in the training group to include in the nomogram. To evaluate the discriminative ability of the nomogram, the area under the receiver operating characteristic curve was utilized. The calibration of the nomogram was examined using the Hosmer-Lemeshow goodness-of-fit test and calibration plots. Additionally, decision curve analysis was performed to determine the clinical utility of the nomogram.

**Results:**

A total of 436 patients with ICH were enrolled in the study. In the training cohort (n = 305), 87 patients had UTI after intracerebral hemorrhage. Multivariate logistic regression analysis demonstrated neutrophil-to-lymphocyte ratio (NLR; OR, 1.357; 95% CI, 1.162–1.899; *p* = 0.036), D-Dimer (OR, 3.050; 95% CI, 1.925–4.856; *p* = 0.023), tumor necrosis factor-*α* (TNF-α; OR, 1.957; 95% CI, 1.670–2.378; *p* < 0.001), and age greater than or equal to 65 years (OR, 2.531; 95% CI, 1.765–3.625; *p* = 0.043) were independent predictors for postoperative UTI and constructed the nomogram. The nomogram demonstrated a high predictive capability with a C-index of 0.865 (95% CI, 0.796–0.935) in the training cohort and 0.867 (95% CI, 0.777–0.958) in the validation cohort. The Hosmer-Lemeshow goodness-of-fit assessment indicated a strong agreement between the predicted probabilities and the observed outcomes for both the training cohort (χ2 = 26.01, df = 8, *p* = 0.136) and the validation cohort (χ2 = 5.652, df = 8, *p* = 0.238). The decision curve analysis demonstrated that the nomogram was markedly effective for predicting UTI in the training cohort and was further validated in the subsequent cohort.

**Conclusion:**

This study presents a new and practical nomogram that uses NLR, D-Dimer, TNF-*α*, and age 65 years or older to effectively predict the risk of postoperative UTI in patients with ICH.

## 1 Introduction

Intracerebral hemorrhage (ICH) is one of the most critical forms of acute stroke, accounting for nearly 50% of stroke-related morbidity and mortality (1). The incidence of ICH has been rising due to an aging population and the increased use of anticoagulants and antiplatelet agents for the treatment of thromboembolic diseases. Although ICH constitutes 6.5 to 19.6% of all strokes, it is associated with a high rate of mortality and morbidity; the 30-day mortality rate ranges from 35 to 52%, and only 20% of survivors are able to live independently (2). Surgery remains one of the primary clinical modalities for treating ICH. While surgical intervention has demonstrated a certain degree of therapeutic efficacy in patients with ICH, physiological dysfunction may persist following extensive and intricate treatment. This is attributable to the high-risk nature of the surgery, potential postoperative complications, and the variability in patient conditions during the postoperative period (3).

Urinary Tract Infection (UTI) is a condition characterized by the proliferation of numerous pathogens within the urinary tract, resulting in inflammation of the mucosal tissues and subsequent uropathy (4). UTIs are a common complication among individuals who have experienced a stroke and are regarded as a substantial risk factor for adverse outcomes. The existing literature indicates that the prevalence of UTI in stroke patients varies between 14.2 and 34.7% (5–7). Furthermore, patients suffering from intracranial hemorrhage exhibit a mortality rate of 29% within 1 year (8), with the occurrence of urinary tract infections in this subgroup ranging from 15.1 to 26.1% (9, 10). The occurrence of urinary tract infection not only increases the chance of other postoperative associated infections, prolongs the patient’s hospital stay, increases the cost of treatment of ICH, increases nursing workload, and reduces the rehabilitation effect, but also may lead to disease deterioration, which imposes a heavy burden on the family and society (11, 12). Therefore, an in-depth understanding of the risk factors for postoperative urinary tract infection in patients with ICH can effectively reduce the risk of postoperative urinary tract infection in patients, make timely and effective preventive and therapeutic measures, and further improve the probability of patient recovery.

Current studies reported (13, 14) in the literature on postoperative urinary tract infections in patients with ICH have primarily focused on traditional risk factors such as age, gender, and D-dimer. However, these studies have largely been limited to identifying independent risk factors for postoperative UTIs. This approach falls short in accurately predicting the potential risks of postoperative UTIs in ICH patients. In contrast, our study incorporates both traditional risk factors and novel inflammatory markers, such as the neutrophil-to-lymphocyte ratio (NLR) and tumor necrosis factor-alpha (TNF-*α*). These inflammatory markers have been increasingly recognized for their role in postoperative complications, including UTIs, and provide additional predictive value beyond traditional factors. Each patient should be assessed individually in clinical practice, integrating multiple parameters and accordingly predicting the risk of developing urinary tract infections after ICH, but such integration of parameters is currently lacking in clinical practice. The nomogram is a graphical statistical tool that can assess and calculate the probability of a specific clinical outcome for patients by using a continuous score. It has been increasingly utilized as a predictive method in stroke in recent years (15, 16). Unlike previous ICH prognosis prediction models that primarily integrate indicators such as the Glasgow Coma Scale (GCS) and hemorrhage volume, our study aims to identify independent predictors associated with postoperative UTI in patients with ICH. We have developed and validated a nomogram that integrates a comprehensive set of clinical data and laboratory variables, including novel inflammatory markers. This approach not only enhances the accuracy of UTI prediction but also provides a more holistic assessment of risk factors.

## 2 Materials and methods

### 2.1 Study design and subjects

**Figure 1:**
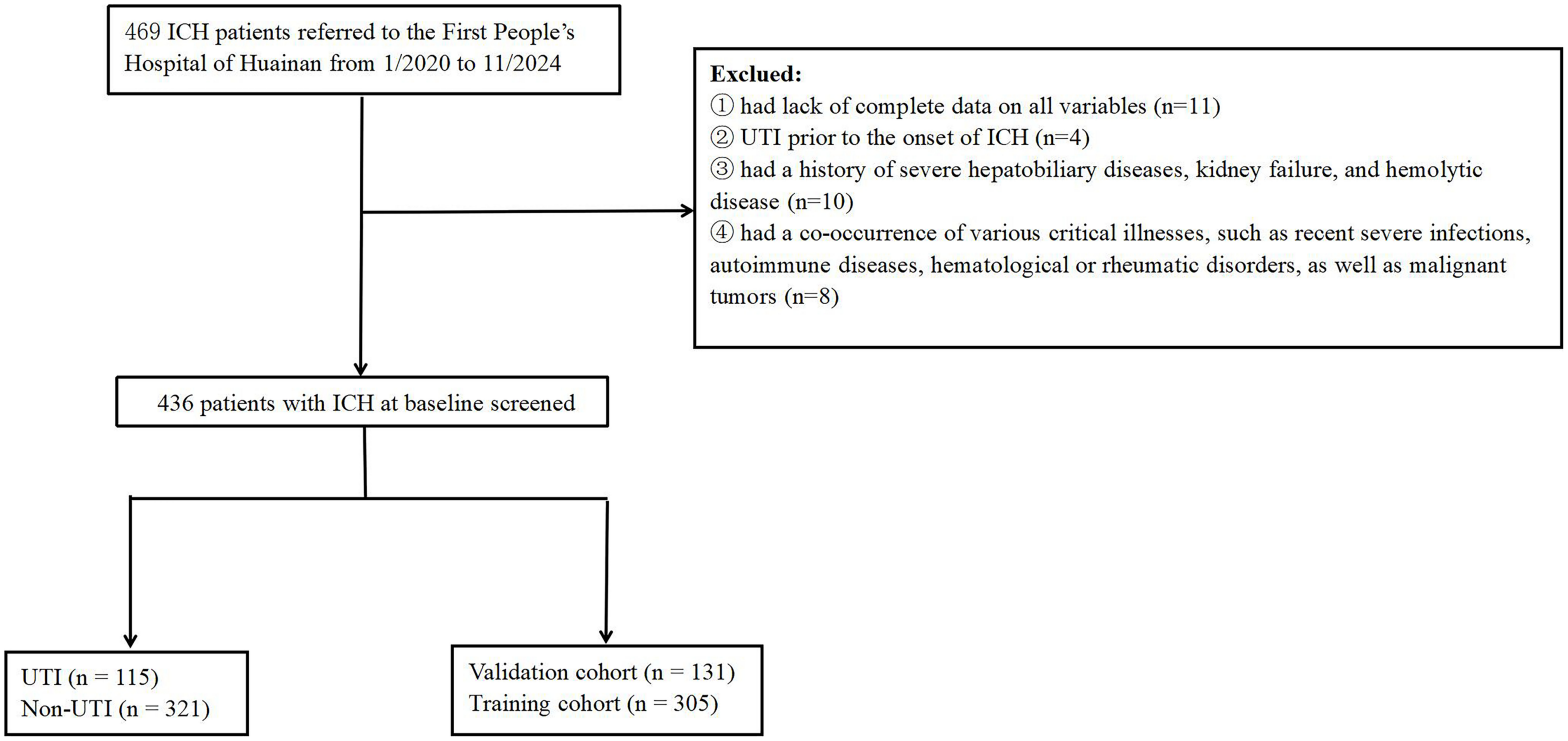
Flowchart of participant selection. ICH, intracerebral hemorrhage; UTI, urinary tract infection.

Our investigation was structured as a single-center prospective cohort study, carried out at the First People’s Hospital of Huainan from January 2020 to November 2024. This study was approved by the ethics committee of First People’s Hospital of Huainan, (approval no. of ethics committee: HN-IRB20200120). In accordance with the principles set forth in the Declaration of Helsinki, all participants provided their written informed consent. Patients with ICH were consecutively recruited in this study (*n* = 469). The identification of UTI was established during the hospital stay following surgical intervention when patients exhibited isolated acute dysuria, or presented with fever accompanied by at least one of the subsequent symptoms: increased urinary frequency, urgency, or retention; incontinence; visible hematuria; alterations in urine properties; or tenderness in the suprapubic region or costovertebral angle. This diagnosis was further supported by the detection of ≥10^3 colony-forming units of any organism in the urine culture (17, 18). Meanwhile, the healthy control group consisted of the age and sex matched individuals who came to our hospital for healthy examination between May 2020 and June 2024. Controls (100 healthy individuals) were composed of 60 males and 40 females. The inclusion criteria were as follows: (1) must be over 18 years of age and sign informed consent; (2) ICH was confirmed by clinical symptoms and computed tomography (CT); (3) patients underwent surgical treatment for ICH. Participants were not included if they: (1) had lack of complete data on all variables; (2) experienced a UTI, prior to the occurrence of ICH; (3) had a history of severe hepatobiliary diseases, kidney failure, and hemolytic disease; (4) had a combination with other serious urological diseases; (5) had a co-occurrence of various critical illnesses, such as recent severe infections, advanced liver and kidney conditions, autoimmune diseases, hematological or rheumatic disorders, as well as malignant tumors. All enrolled patients were divided into the training cohort and validation cohort based on the pre-seeded random number (123) generator in R software (version 4.4.2). Finally, the patients were randomly divided into training (*n* = 305) and validation (*n* = 131) cohorts based on the ratio of 7:3. At last, the study included 436 cases with ICH in total (Figure 1).

### 2.2 Clinical data collection

Baseline characteristics include demographic data (including age, gender, education years, married), clinical data (including the history of hypertension, diabetes mellitus, smoking, and drinking) were collected for patients at admission.

### 2.3 Blood collection and laboratory test

Venous blood was drawn from patients the next morning after admission. We measured white blood cell (WBC) counts, neutrophil to-lymphocyte ratio (NLR), erythrocyte count, hemoglobin (Hb), fibrinogen, D-Dimer, glucose (G), prealbumin (PA), triglyceride (TC), triglycerides (TG), c-reactive protein (CRP), high-density lipoprotein (HDL), low-density lipoprotein (LDL), apolipoprotein A (ApoA), and apolipoprotein B (ApoB), and tumor necrosis factor-*α* (TNF-α). The plasma TNF-α concentrations were measured using enzyme-linked immunosorbent assay (ELISA) kits (model: PR108125, purchased from Shanghai Bohu Biotechnology Co., Ltd.). Moreover, intraoperative data (intra-operative time) and postoperative data (tracheotomy and tracheotomy) were also collected. Fresh mid-stream urine samples were collected from all patients during their first morning urination. These samples were immediately placed in sealed urinalysis cups. Urine dry chemistry analysis and urine sediment examination were conducted within 1 h of collection to ensure the accuracy and reliability of the results. The Roche urine dry chemistry analyzer (URISYS-2400) and the automatic urine sediment analyzer (SC7KBX) from Kobelco, Germany, were selected as the primary instruments for the analysis. The corresponding reagents specifically designed for these instruments were also utilized to ensure optimal performance and accuracy of the results.

### 2.4 Statistical analysis

Statistical analyses were conducted utilizing the R statistical software (version 4.4.2; R Foundation for Statistical Computing, Vienna, Austria). Categorical variables were reported as frequencies (n) and percentages (%), while continuous variables were presented as either means with standard deviations (SD) or medians accompanied by interquartile ranges. To evaluate the differences in baseline characteristics across the groups, independent sample t-tests were utilized for continuous variables that met parametric assumptions, while the Mann–Whitney U tests were employed for non-parametric continuous variables. For categorical variables, the chi-squared test or Fisher’s exact test was applied, depending on the suitability of the data. Variables that exhibited a *p*-value of less than 0.05 in the univariate analysis were subsequently incorporated into the multivariate logistic regression analysis. The assessment of collinearity among the variables was conducted using the variation inflation factors, with a threshold of greater than 5 deemed significant. Ultimately, the odds ratio (OR) along with the 95% confidence interval (CI) for each variable was determined through the application of multivariate logistic regression analysis. A new nomogram was developed to create a predictive model, utilizing significant predictors of UTI identified through multivariate logistic regression analysis employing the forward selection method. The area under the receiver operating characteristic (ROC) curve was employed to assess the discriminatory performance of the nomogram in both the training and validation groups. The calibration efficacy of the nomogram was evaluated in both the training cohort and the validation cohort through the application of the Hosmer-Lemeshow goodness-of-fit test, alongside the generation of a calibration plot utilizing 1,000 bootstrap resamples. This analysis provided insights into the agreement between the predicted probabilities and the observed outcomes.

## 3 Results

Relative to patients in non-UTI group after ICH, those in UTI group presented higher proportions of age ≥65y (*p* = 0.002), higher NLR (*p* = 0.018), higher TNF-*α* (*p* = 0.011), higher G (*p =* 0.002), as well as higher D-Dimer (*p <* 0.001). The basic characteristics of the 436 patients were presented in Table 1. Indeed, the results also indicated that the TNF-α levels in the UTI group were significantly higher than those in the non-UTI group and the healthy control group (15.90 [15.39–16.40] vs. 28.99 [9.68–9.93] and 3.23 [1.02–4.21], *p* < 0.001).

**Table 1:** Demographic and clinical characteristics of patients without and with UTI.

	Non-UTI (*n* = 321)	UTI (*n* = 115)	Health controls (*n* = 100)	*p*-value
Demographic characteristics				
Males, n (%)	191 (59.50)	69 (60.00)	60 (60)	0.926
Married, n (%)	306 (95.33)	108 (93.91)	100 (100)	0.729
Education years, median (IQR)	5.00 (3.00–8.00)	6.00 (4.00–8.00)		0.110
Age ≥ 65y	171 (53.27)	81 (70.43)	56 (56)	0.006
Vascular risk factors (%)				
Hypertension, n (%)	205 (63.86)	72 (62.61)		0.899
Diabetes mellitus, n (%)	88 (27.41)	41 (35.65)		0.123
Current smoking, n (%)	97 (30.22)	36 (31.30)		0.921
Alcohol consumption, n (%)	111 (34.58)	36 (31.30)		0.601
Laboratory parameters (IQR)				
NLR, median (IQR)	2.95 (2.70–3.21)	3.62 (3.06–4.02)		<0.001
WBC, ×109/L, median (IQR)	6.36 (5.58–7.50)	6.37 (5.55–7.53)		0.777
CRP, mg/L, median (IQR)	5.77 (5.13–6.40)	5.97 (5.00–6.63)		0.621
PA, mg/L, median (IQR)	197.43 (182.99–245.00)	190.12 (186.78–257.98)		0.538
D-Dimer, mg/L, median (IQR)	1.61 (1.36–1.71)	1.68 (1.59–1.89)		<0.001
Fibrinogen,g/L, median (IQR)	3.36 (3.17–3.44)	3.42 (3.00–3.47)		0.074
G, mmol/L, median (IQR)	5.20 (4.70–6.30)	5.80 (5.10–6.80)		0.002
TNF-α, pg./mg, median (IQR)	9.68 (9.25–9.93)a	15.90 (15.39–16.40)b	3.23 (1.02–4.21)	<0.001
Hb, g/L, median (IQR)	139.00 (134.00–144.00)	142.00 (134.00–145.00)		0.687
Erythrocyte count, median (IQR)	4.78 (4.51–5.21)	4.89 (4.51–5.22)		0.466
TG, mmol/L, median (IQR)	1.33 (0.94–1.93)	1.37 (0.99–1.91)		0.975
TC, mmol/L, median (IQR)	4.41 (3.74–5.16)	4.60 (3.76–5.32)		0.317
HDL, mmol/L, median (IQR)	1.01 (0.86–1.21)	1.03 (0.86–1.29)		0.423
LDL, mmol/L, median (IQR)	2.51 (1.97–3.18)	2.62 (2.04–3.16)		0.240
APA, g/L, median (IQR)	1.26 (1.08–1.43)	1.27 (1.12–1.44)		0.616
APB, g/L, median (IQR)	0.85 (0.69–1.00)	0.88 (0.72–1.07)		0.150
Hematoma location, n (%)				
Cerebellum, n (%)	34 (10.59)	15 (13.04)		0.588
Basal ganglia, n (%)	122 (38.01)	35 (30.43)		0.181
Intraventricular hemorrhage, n (%)	17 (5.30)	12 (10.43)		0.903
Bleeding from other locations, n (%)	138 (42.99)	53 (46.09)		0.642
Subarachnoid hemorrhage, n (%)	54 (16.82)	11 (9.57)		0.085
Tracheotomy, n (%)	85 (26.48)	40 (34.78)		0.117
Epidural tube, n (%)	94 (29.28)	45 (39.13)		0.068
External ventricular drain, n (%)	20 (6.23)	8 (6.96)		0.959
Intra-operative time, median (IQR)	214.10 (189.40–248.60)	221.50 (198.15–251.50)		0.283

WBC, white blood cell; NLR, neutrophil to-lymphocyte ratio; G, glucose; PA, prealbumin; TC, triglyceride; TG, triglycerides; CRP, c-reactive protein; HDL, high-density lipoprotein; LDL, low-density lipoprotein; ApoA, apolipoprotein A; ApoB, apolipoprotein B; TNF-α, tumor necrosis factor-α. ^a^*p* < 0.001 compared to UTI; ^b^*p* < 0.001 compared to healthy control.

A total of 436 patients participated in the study, which were subsequently categorized into a training cohort consisting of 305 individuals and a validation cohort comprising 131 individuals for subsequent analysis. Comprehensive details regarding the baseline characteristics of all participants are presented in Table 1. There were no notable differences in the variables between the training cohort and the validation cohort. As shown in Table 2, 87 (28.25%) were Postoperative UTI in the training cohort. The univariate analysis revealed that NLR, Age ≥ 65y, TNF-*α*, and D-Dimer were related to non-UTI (*p* < 0.05). No significant statistical collinearity was observed among all variables (Supplementary Table 1). After multivariate logistic regression analysis, TNF-α (OR, 1.957; 95% CI, 1.670–2.378; *p* < 0.001), NLR (OR, 1.357; 95% CI, 1.162–1.899; *p* = 0.036), age ≥ 65 years (OR, 2.531; 95% CI, 1.765–3.625; *p* = 0.043), and D-dimer (OR, 3.050; 95% CI, 1.925–4.856; *p* = 0.023) were identified as independent predictors for the occurrence of UTI in patients following ICH, as shown in Table 3. In the validation cohort, we found significant differences in NLR, Age ≥ 65y, TNF-*α*, and D-Dimer between the UTI and non-UTI groups (Supplementary Table 1).

**Table 2:** Demographics and clinical characteristics of the training and validation cohort.

	All patients (*n* = 436)	Validation cohort (*n* = 131)	Training cohort (*n* = 305)	*p*-value
Demographic characteristics				
Males, n (%)	260 (60)	80 (61)	180 (59)	0.769
Married, n (%)	414 (95)	127 (97)	287 (94)	0.314
Education years, median (IQR)	5 (3–8)	5 (3–7)	5 (3–8)	0.859
Age≥65y	252 (58)	77 (59)	175 (57)	0.868
Vascular risk factors (%)				
Hypertension, n (%)	277 (64)	91 (69)	186 (61)	0.114
Diabetes mellitus, n (%)	129 (30)	41 (31)	88 (29)	0.69
Current smoking, n (%)	133 (31)	45 (34)	88 (29)	0.303
Alcohol consumption, n (%)	147 (34)	52 (40)	95 (31)	0.105
Laboratory parameters (IQR)				
NLR, median (IQR)	3.01 (2.77–3.58)	3.05 (2.78–3.65)	2.99 (2.74–3.58)	0.095
WBC, ×109/L, median (IQR)	6.36 (5.57–7.5)	6.38 (5.62–7.61)	6.36 (5.57–7.44)	0.575
CRP, mg/L, median (IQR)	5.81 (5.07–6.49)	5.85 (5.00–6.35)	5.79 (5.12–6.57)	0.276
PA, mg/L, median (IQR)	195.23 (183.2–257.98)	197.45 (184.39–251.32)	194.55 (183.12–257.98)	0.51
D-Dimer, mg/L, median (IQR)	1.63 (1.39–1.73)	1.63 (1.38–1.74)	1.63 (1.39–1.72)	0.438
Fibrinogen, g/L, median (IQR)	3.39 (3.15–3.44)	3.42 (3.17–3.47)	3.39 (3.12–3.44)	0.105
G, mmol/L, median (IQR)	5.4 (4.8–6.6)	5.3 (4.7–6.4)	5.4 (4.8–6.64)	0.347
TNF-α, pg./mg, median (IQR)	9.79 (9.44–10.61)	9.77 (9.29–10.66)	9.79 (9.45–10.6)	0.753
Hb, g/L, median (IQR)	140 (134–45)	140 (131–144)	140 (134–145)	0.286
Erythrocyte count, median (IQR)	4.78 (4.51–5.21)	4.78 (4.52–5.26)	4.78 (4.48–5.21)	0.238
TG, mmol/L, median (IQR)	1.34 (0.94–1.92)	1.37 (0.94–2.00)	1.33 (0.95–1.87)	0.346
TC, mmol/L, median (IQR)	4.48 (3.74–5.22)	4.41 (3.77–5.26)	4.51 (3.73–5.21)	0.976
HDL, mmol/L, median (IQR)	1.02 (0.86–1.24)	0.99 (0.81–1.17)	1.03 (0.87–1.26)	0.077
LDL, mmol/L, median (IQR)	2.54 (1.98–3.18)	2.6 (1.96–3.2)	2.51 (2.00–3.16)	0.681
APA, g/L, median (IQR)	1.26 (1.09–1.43)	1.26 (1.06–1.37)	1.26 (1.12–1.46)	0.053
APB, g/L, median (IQR)	0.86 (0.69–1.01)	0.85 (0.67–1.03)	0.86 (0.7–1.01)	0.85
Hematoma location, n (%)				
Cerebellum, n (%)	49 (11)	17 (13)	32 (10)	0.557
Basal ganglia, n (%)	157 (36)	43 (33)	114 (37)	0.424
Intraventricular hemorrhage, n (%)	29 (7)	11 (8)	18 (6)	0.454
Bleeding from other locations, n (%)	191 (44)	53 (40)	138 (45)	0.413
Subarachnoid hemorrhage, n (%)	65 (15)	19 (15)	46 (15)	0.993
Tracheotomy, n (%)	125 (29)	41 (31)	84 (28)	0.497
Epidural tube, n (%)	139 (31.88)	94 (29.28)	45 (39.13)	0.068
External ventricular drain, n (%)	28 (6)	12 (9)	16 (5)	0.188
Intra-operative time, median (IQR)	216.15 (191.48–249.15)	218.5 (192.05–249.4)	214.6 (191.4–249.1)	0.528

WBC, white blood cell; NLR, neutrophil to-lymphocyte ratio; G, glucose; PA, prealbumin; TC, triglyceride; TG, triglycerides; CRP, c-reactive protein; HDL, high-density lipoprotein; LDL, low-density lipoprotein; ApoA, apolipoprotein A; ApoB, apolipoprotein B; TNF-α, tumor necrosis factor-α.

**Table 3:** Baseline characteristics and logistic regression analysis between the UTI group and non-UTI group in the training cohort.

Variables	Non-UTI group (*n* = 218)	UTI group (*n* = 87)	Univariate logistic regression analysis		Multivariate logistic regression analysis	
			OR (95% CI)	*p* value	OR (95% CI)	*p* value
Demographic characteristics						
Sex, n (%)	137 (59.83)	43 (56.58)	1.143 (0.673–1.929)	0.618		
Married, n (%)	218 (95.20)	69 (90.79)	2.011 (0.716–5.308)	0.165		
Education years, median (IQR)	5.00 (3.00–8.00)	6.00 (4.00–8.00)	1.012 (0.954–1.069)	0.642		
Age, (≥65y), n (%)	130 (56.77)	59 (77.63)	2.643 (1.478–4.934)	0.001	2.531 (1.765–3.625)	0.043
Vascular risk factors (%)						
Hypertension, n (%)	142 (62.01)	44 (57.89)	1.187 (0.697–2.008)	0.524		
Diabetes mellitus, n (%)	60 (26.20)	28 (36.84)	0.609 (0.352–1.063)	0.078		
Current smoking, n (%)	64 (27.95)	24 (31.58)	0.84 (0.482–1.492)	0.545		
Alcohol consumption, n (%)	74 (32.31)	21 (27.63)	1.25 (0.712–2.255)	0.445		
Laboratory parameters (IQR)						
NLR, median (IQR)	2.94 (2.68–3.20)	3.62 (3.04–3.90)	3.938 (2.473–6.554)	0.000	1.357 (1.162–1.899)	0.036
WBC, ×109/L, median (IQR)	6.33 (5.57–7.43)	6.50 (5.54–7.65)	1.09 (0.894–1.327)	0.391		
CRP, mg/L, median (IQR)	5.78 (5.18–6.50)	5.94 (4.97–6.71)	1.006 (0.773–1.303)	0.965		
PA, mg/L, median (IQR)	197.43 (182.11–258.64)	189.12 (185.44–257.98)	0.997 (0.993–1.001)	0.152		
D-Dimer, mg/L, median (IQR)	1.62 (1.37–1.70)	1.67 (1.54–1.86)	5.444 (2.65–12.92)	0.000	3.050 (1.925–4.856)	0.023
Fibrinogen,g/L, median (IQR)	3.36 (3.15–3.42)	3.40 (2.90–3.50)	1.145 (0.699–1.825)	0.574		
G, mmol/L, median (IQR)	5.20 (4.70–6.50)	5.80 (5.10–6.98)	1.103 (0.938–1.292)	0.226		
TNF-α, median (IQR)	9.69 (9.28–9.94)	15.91 (15.39–16.40)	2.101(1.802–2.534)	0	1.957 (1.670–2.378)	0.000
Erythrocyte count, median (IQR)	4.67 (4.48–5.21)	4.92 (4.51–5.29)	1.481 (0.916–2.427)	0.113		
Hb, median (IQR)	139.00 (134.00–145.00)	141.50 (134.00–145.25)	1.002 (0.979–1.026)	0.841		
TG, mmol/L, median (IQR)	1.31 (0.92–1.80)	1.37 (1.01–1.92)	1.04 (0.847–1.247)	0.683		
TC, mmol/L, median (IQR)	4.48 (3.74–5.12)	4.61 (3.68–5.54)	1.09 (0.918–1.289)	0.306		
HDL, mmol/L, median (IQR)	1.02 (0.86–1.23)	1.04 (0.90–1.31)	1.56 (0.979–3.19)	0.149		
LDL, mmol/L, median (IQR)	2.48 (1.98–3.16)	2.60 (2.02–3.21)	1.154 (0.862–1.542)	0.333		
APA, g/L, median (IQR)	1.26 (1.09–1.46)	1.29 (1.16–1.48)	1.422 (0.547–3.632)	0.463		
APB, g/L, median (IQR)	0.85 (0.69–0.98)	0.89 (0.76–1.06)	1.191 (0.641–2.168)	0.529		
Hematoma location, n (%)						
Cerebellum, n (%)	21 (9.17)	11 (14.47)	0.597 (0.278–1.344)	0.195		
Basal ganglia, n (%)	89 (38.86)	25 (32.89)	1.297 (0.756–2.267)	0.352		
Intraventricular hemorrhage, n (%)	11 (4.80)	7 (9.21)	0.497 (0.188–1.398)	0.165		
Bleeding from other locations, n (%)	100 (43.67)	38 (50.00)	0.775 (0.46–1.305)	0.337		
Subarachnoid hemorrhage, n (%)	39 (17.03)	7 (9.21)	2.023 (0.914–5.131)	0.104		
Tracheotomy, n (%)	57 (24.89)	27 (35.53)	0.601 (0.346–1.057)	0.074		
Epidural tube, n (%)	71 (31.00)	30 (39.47)	1.451 (0.842–2.48)	0.175		
External ventricular drain, n (%)	14 (6.11)	2 (2.63)	2.409 (0.653–15.57)	0.252		
Intra-operative time, median (IQR)	213.90 (191.40–245.10)	219.65 (194.95–254.50)	1.002 (0.996–1.007)	0.562		

WBC, white blood cell; NLR, neutrophil to-lymphocyte ratio; G, glucose; PA, prealbumin; TC, triglyceride; TG, triglycerides; CRP, c-reactive protein; HDL, high-density lipoprotein; LDL, low-density lipoprotein; ApoA, apolipoprotein A; ApoB, apolipoprotein B; TNF-α, tumor necrosis factor-α.

**Figure 2:**
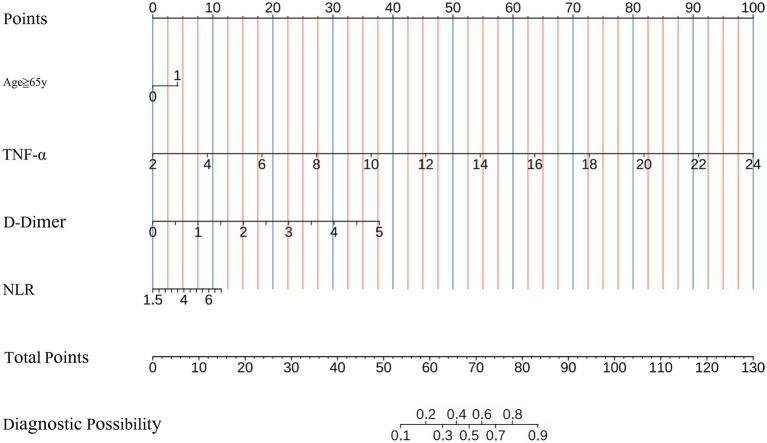
Nomogram for predicting the probability of postoperative UTI in ICH patients based on NLR, Age≥65y, TNF-*α*, and D-Dimer levels. UTI, urinary tract infection; ICH, intracerebral hemorrhage; NLR, neutrophil to-lymphocyte ratio, TNF-α, tumor necrosis factor-α.

All independent predictors for UTI after ICH were used to construct the novel nomogram (Figure 2). The nomogram consisted of the preliminary value of predictors, preliminary score range (0–130), total score, and probability of UTI. Drawing a line downward from the preliminary value to the corresponding preliminary score, and then summed all the preliminary scores to obtain a total score. Finally, the percentage corresponding to the total score was the individual probability of UTI after ICH.

**Figure 3:**
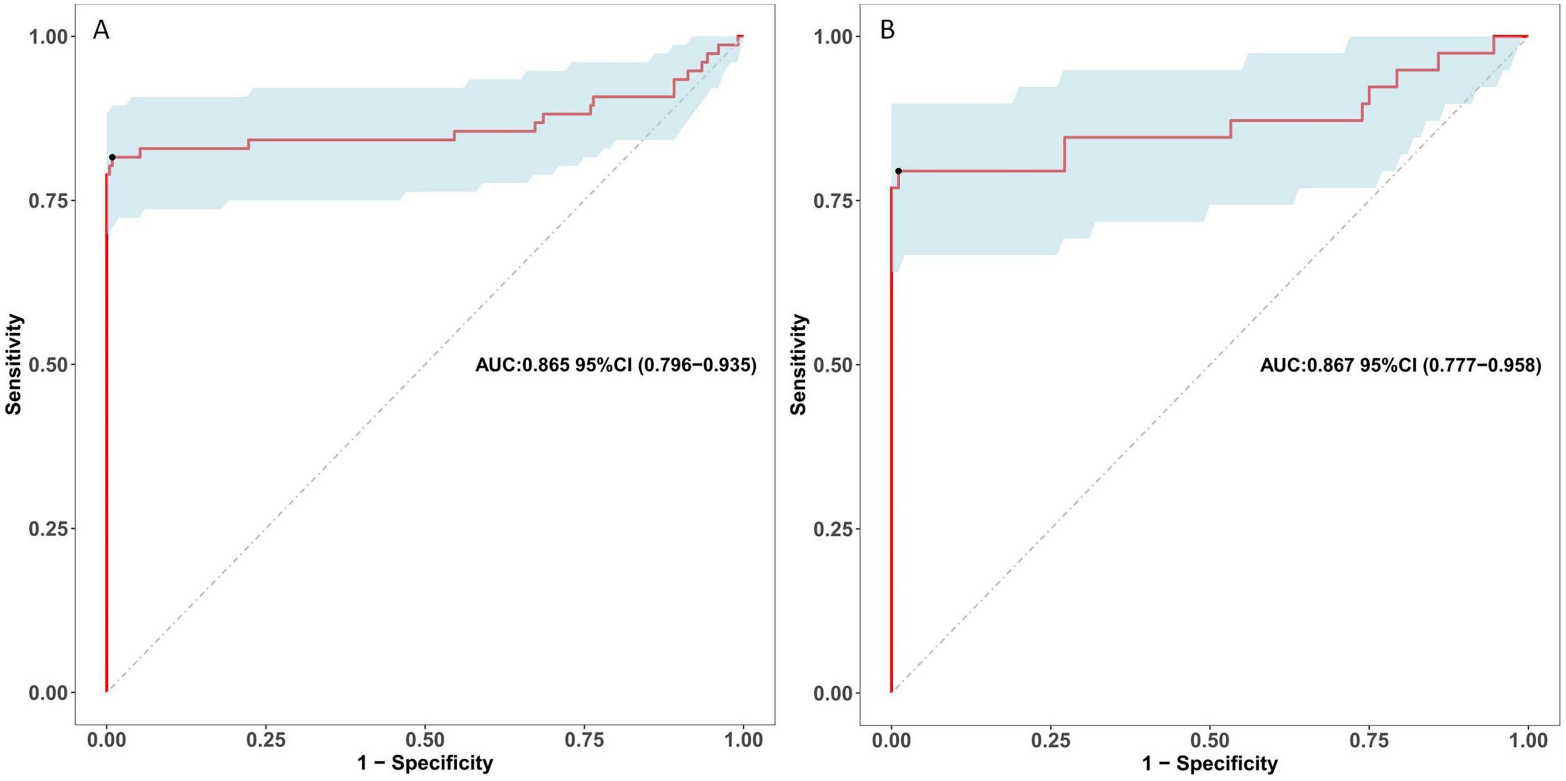
Receiver operating characteristic curves for risk of postoperative UTI in ICH patients. **(A)** Presents the ROC curve for the training dataset. **(B)** Illustrates the ROC Curve for the validation dataset. The sensitivity and specificity associated with various risk thresholds of the predictive model are illustrated in a graphical representation. UTI, urinary tract infection; ICH, intracerebral hemorrhage; ROC, receiver operating characteristic.

**Figure 4:**
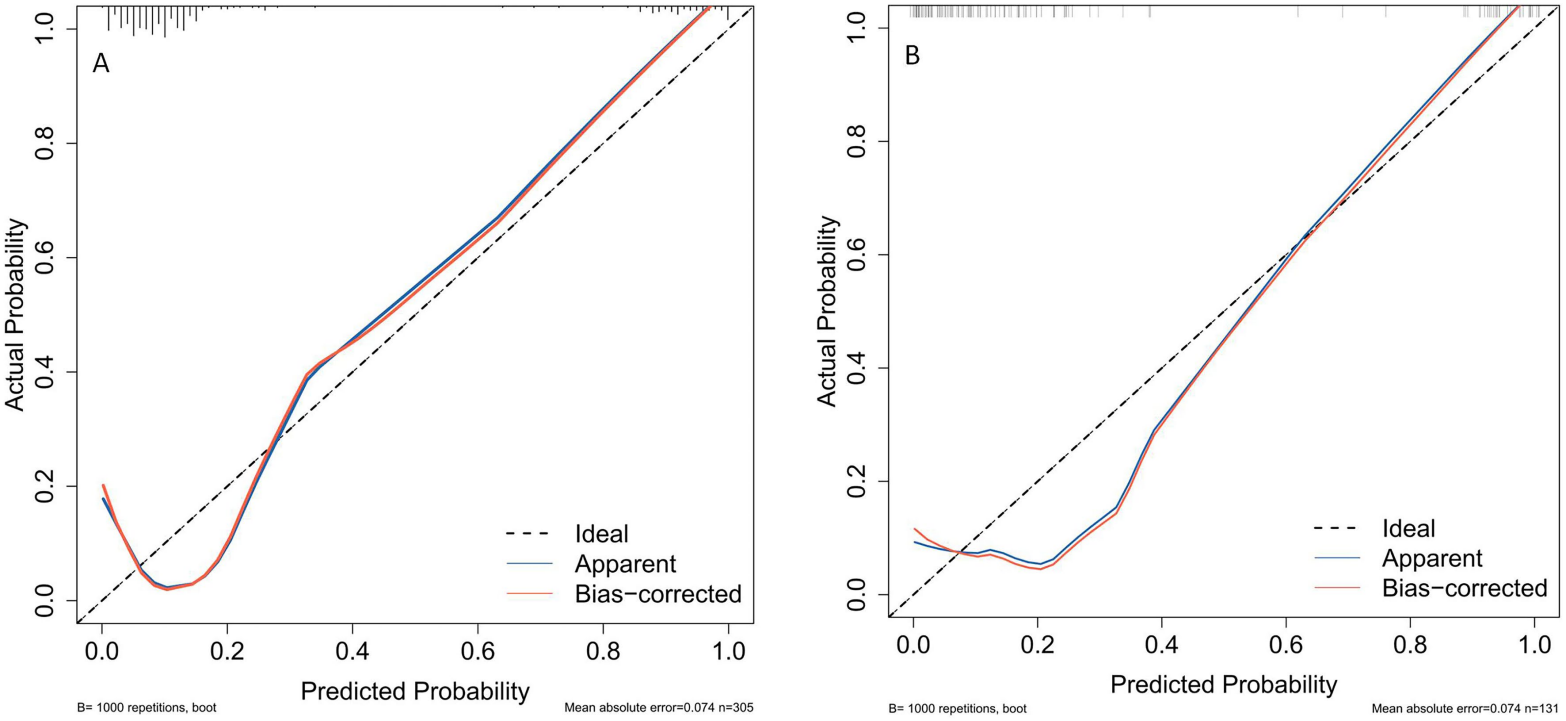
Calibration plots for predicting postoperative UTI in ICH patients for both the training cohort **(A)** and the validation cohort **(B)**. UTI, urinary tract infection; ICH, intracerebral hemorrhage.

**Figure 5:**
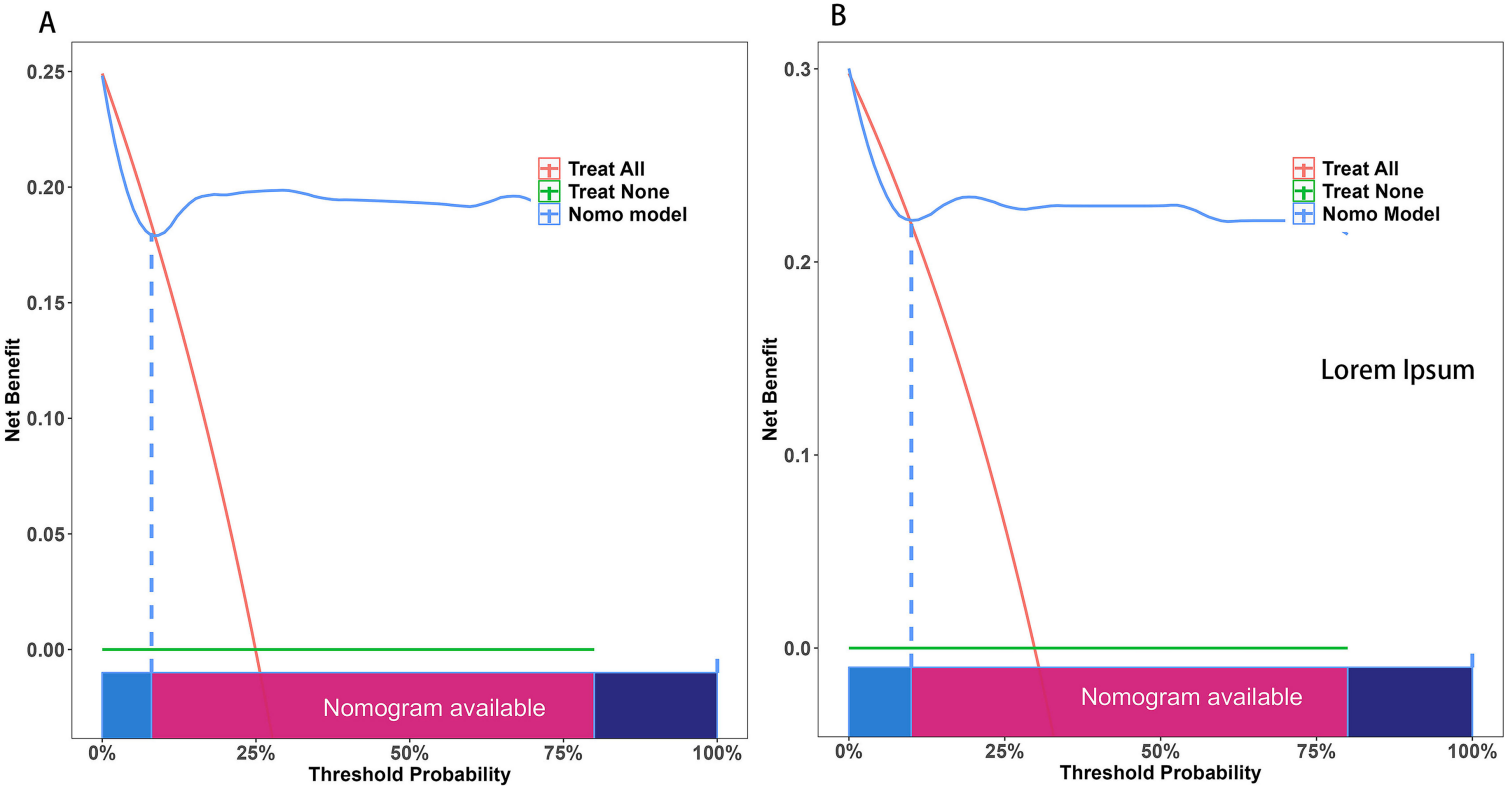
The Decision Curve Analysis (DCA) was performed on the nomogram designed to forecast postoperative UTI following ICH within both the training cohort **(A)** and the validation cohort **(B)**. The x-axis illustrates the probability threshold. The y-axis indicates the net benefit. The green line indicates that all patients tested negative and did not receive any treatment, resulting in a net benefit of zero. The red line indicates that every patient undergoing surgical intervention is expected to experience a UTI. The blue line represents the overall advantage provided by the nomogram. UTI, urinary tract infection.

The ROC curve was employed to assess the discriminative capability of the nomogram, revealing a moderate level of predictive accuracy within the training cohort (AUC, 0.865; 95% CI, 0.796–0.935) as illustrated in Figure 3A, and in the validation cohort (AUC, 0.867; 95% CI, 0.777–0.958) as depicted in Figure 3B. The Hosmer-Lemeshow goodness-of-fit assessment indicated a strong agreement between the predicted probabilities and the observed outcomes for both the training cohort (χ2 = 26.01, df = 8, *p* = 0.136) and the validation cohort (χ2 = 5.652, df = 8, *p* = 0.238). The calibration plot demonstrated a notable level of predictive precision of the nomogram for forecasting UTI subsequent to ICH, as observed in both the training cohort (Figure 4A) and the validation cohort (Figure 4B). As illustrated in Figure 5, the decision curve analysis revealed that within the training set, threshold probabilities from 8.6 to 85% (Figure 5A), and within the validation set, from 10 to 100%(Figure 5B), the application of the nomogram for forecasting Postoperative UTI yielded a superior net benefit compared to the “treat all” or “treat none” approaches. This finding underscores the clinical relevance of utilizing the nomogram.

## 4 Discussion

In the current investigation, a detailed nomogram was developed utilizing variables including the NLR, Age ≥ 65y, TNF-*α*, and D-Dimer levels. This model aims to effectively forecast the occurrence of postoperative UTI in patients suffering from intracranial hemorrhage. The outstanding ability of the nomogram to discriminate and calibrate was clearly demonstrated in the training cohort and further validated through external assessments. Ultimately, the DCA, which serves as a specialized instrument for assessing the clinical utility of a nomogram, indicated that our nomogram holds significant potential for forecasting postoperative UTI in a clinical practice. A significant advantage of our model is its foundation on pre-treatment parameters, all of which can be acquired prior to the commencement of any therapeutic measures. This not only increases its clinical significance but also provides healthcare professionals, patients, and their families with a practical guideline, enabling a well-informed decision-making process from the initial stages.

Both univariate and multivariate analyses indicated that increased age correlated with a higher incidence of postoperative UTI in this investigation. This finding aligns with earlier research that posits age over 65 years as a contributing factor for UTI in patients suffering from intracranial hemorrhage (19, 20). With advancing age, the anatomical barriers and physiological functions of the urinary system experience a progressive decline. This deterioration can result in various complications, including metabolic and immune dysfunctions, alterations in urine pH and glucose levels, as well as increased adhesion of pathogens to the bladder wall, all of which may elevate the risk of UTI in older patients. Consequently, it is imperative to consider the influence of aging on the occurrence of postoperative UTI in individuals who have suffered an intracranial hemorrhage.

The NLR is an indicator of inflammation that has received much attention in recent years, and studies have demonstrated the role of the NLR in assessing inflammatory status and cell-mediated immunity (21). Currently, the literature reports that NLR has been used in the evaluation of diseases such as oncology, cardiovascular diseases, inflammation, and viruses (22–25). The increased NLR can assist clinicians in the early identification of urinary tract infections and help clinicians to determine the entry of urinary tract infection pathogens into the bloodstream, which is of high clinical auxiliary diagnostic value. A biomarker that is readily available in the clinic, the ease of measurement and low cost of NLR compared to other prognostic biomarkers have made it a highly sought-after indicator of inflammation in recent years. A study in the Korea suggested that the NLR may help emergency physicians to predict bacteremia in older adults with UTI visiting the emergency department (26). In contrast, our study found that univariate and multivariate analyses showed that the greater the NLR, the higher the incidence of postoperative urinary tract infections in patients with ICH.

The results of our study also demonstrated that elevated D-dimer is an independent risk factor for postoperative UTI in patients after ICH. Mu et al. (14) demonstrated that D-dimer was significantly elevated in patients with UTI from ICH, and that D-dimer increased the risk of UTI in patients by 1.403 times. D-dimers are soluble fibrin degradation products, and increased concentrations of D-dimers indicate enhanced fibrinolytic activity of fibrin *in vivo*, which may be a predictor of deep vein thrombosis (27). Fibrinolytic activity is significantly increased in patients with bacterial infections and sepsis and is considered a marker of inflammation in urinary tract infections (28). Elevated D-dimer in patients with urinary tract infections may be a result of the mutual promotion of inflammation and coagulation (29). Therefore, clinical testing of D-dimer levels is not only helpful in determining the risk of thrombosis, but also in understanding urinary tract infections. Appropriate use of anticoagulant and antifibrinolytic drugs, depending on the specific situation, can prevent and block the activation of the coagulation pathway at an early stage, and help to reduce or delay the severity of urinary tract infections. Consequently, the finding that D-dimers increase the risk of urinary tract infections may provide a new rationale for the use of anticoagulant and antifibrinolytic drugs in the treatment of ICH, but further studies are needed. TNF-*α* is an important inflammatory cytokine that plays a key role in the body’s immune response, especially against bacterial infections (30). When the body is infected with bacteria, such as a urinary tract infection, the immune system releases TNF-*α* to promote an inflammatory response (31). Following a bacterial infection, various cells in the body, such as macrophages and monocytes, quickly release numerous cytokines, including TNF-*α*, that trigger an inflammatory response in the host (32). The immune system of patients after ICH may be more sensitive due to surgical stress, physical recovery, and other factors, such as the occurrence of UTI, and increased levels of TNF-*α*. Therefore, elevated levels of TNF-α in patients after ICH can be viewed as an immune response to urinary tract infection, a response designed to fight infection but also a marker of the presence of infection. In patients with chronic kidney disease, elevated serum TNF-*α* levels demonstrate a significant association with increased hospitalization risk due to UTI, suggesting that TNF-*α* may exacerbate infection susceptibility through impaired immune regulation (33). Complementing these findings, a vaccine development study targeting recurrent UTIs revealed that therapeutic efficacy was mediated through macrophage activation, which stimulates TNF-*α* secretion to enhance phagocytic clearance of *Escherichia coli*. This mechanistic evidence highlights TNF-α’s dual regulatory role in UTI pathophysiology - while excessive production may drive immunopathological responses in CKD patients, its controlled release appears crucial for orchestrating innate immune defenses against uropathogens (34). Compared with other inflammatory markers, TNF-*α* has some unique advantages. For example, it has a relatively high specificity for infection-related inflammation (35). While other markers like CRP may also be elevated in various inflammatory conditions, TNF-α is more closely associated with the presence of active infection (36). This makes it a more reliable predictor of UTI in the context of ICH. In summary, the elevated TNF-α levels in ICH patients not only indicate an immune response to UTI but also serve as a significant predictor due to its early appearance, high specificity for infection-related inflammation, and central role in the immune response. These characteristics make TNF-α a valuable marker for monitoring and managing UTI in ICH patients compared to other inflammatory markers.

Our study presents several limitations that merit attention. Firstly, it is important to note that this investigation was conducted as a small-sample, single-center retrospective cohort study. To enhance the statistical robustness of our findings, we selected only those variables that exhibited *p* values less than 0.05 in univariate analyses as potential candidates for multivariate regression analyses. Secondly, the nomogram has not undergone validation within an external cohort. Consequently, it is imperative to conduct a multicenter prospective study to assess the nomogram’s applicability prior to its implementation in clinical settings. Thirdly, despite our thorough efforts to consider various factors in developing our predictive model, we recognize that some unmeasured baseline variables might still affect the likelihood of postoperative UTI after intracranial hemorrhage. These unaccounted factors could include the National Institute of Health Stroke Scale (NIHSS) score, the Glasgow Coma Scale (GCS) score, the use of urinary catheters, and the administration of immunosuppressive medications (e.g., steroids).

## 5 Conclusion

This research introduces an innovative and functional nomogram that utilizes the NLR, age of 65 years or older, TNF-*α*, and D-Dimer concentrations to accurately forecast the likelihood of developing postoperative UTI following intracranial hemorrhage. The calibration and discrimination of the nomogram were assessed through internal validation. This nomogram can be useful for predicting the probability of postoperative UTI after ICH, primarily supporting early clinical decision-making. While the potential for using these indicators to guide the dynamic adjustment of anti-infective therapy postoperatively is an important consideration, further studies are needed to analyze the dynamic changes in these indicators during therapy and to provide evidence linking indicator changes to therapy adjustment outcomes.

## Data Availability

The original contributions presented in the study are included in the article/Supplementary material, further inquiries can be directed to the corresponding author.
