# Supplementary material for: Construction and validation of a risk prediction model for postoperative urinary tract infection in intracranial hemorrhage patients
Source: Front Med (Lausanne). 2025 Nov 21;12:1552191. doi: 10.3389/fmed.2025.1552191 (PMC12678245; doi:10.3389/fmed.2025.1552191)
Supplement: Supplementary file 1 [file Table_1.DOCX]

**Supplementary**

**Comparison of baseline characteristics of intracerebral hemorrhage patients between UTI group and non-UTI group in the validation cohort.**

|  | All patients(n=131) | non-UTI group (n=92) | UTI group (n=39) | P-value |
| --- | --- | --- | --- | --- |
| **Demographic characteristics** |  |  |  |  |
| Males, n (%) | 80 (61) | 54 (59) | 26 (67) | 0.51 |
| Married, n (%) | 127 (97) | 88 (96) | 39 (100) | 0.317 |
| Education years, median (IQR) | 5 (3, 7) | 5 (3, 7) | 6 (4, 7) | 0.53 |
| Age≥65y | 63(46) | 31(33) | 32(82) | ＜0.001 |
| **Vascular risk factors (%)** |  |  |  |  |
| Hypertension, n (%) | 91 (69) | 63 (68) | 28 (72) | 0.865 |
| Diabetes mellitus, n (%) | 41 (31) | 28 (30) | 13 (33) | 0.904 |
| Current smoking, n (%) | 45 (34) | 33 (36) | 12 (31) | 0.718 |
| Alcohol consumption, n (%) | 52 (40) | 37 (40) | 15 (38) | 1.000 |
| **Laboratory parameters (IQR)** |  |  |  |  |
| NLR, median (IQR) | 3.05 (2.78, 3.65) | 2.97 (2.72, 3.28) | 3.62 (3.08, 4.35) | < 0.001 |
| WBC, ×10^9^/L, median (IQR) | 6.38 (5.62, 7.61) | 6.43 (5.64, 7.78) | 6.12 (5.58, 7.32) | 0.554 |
| CRP, mg/L, median (IQR) | 5.69 ± 1.07 | 5.59 ± 0.95 | 5.94 ± 1.29 | 0.128 |
| PA, mg/L, median (IQR) | 197.45 (184.39, 251.32) | 197.43 (182.99, 237.02) | 221 (188.83, 258.05) | 0.255 |
| D-Dimer, mg/L, median (IQR) | 1.63 (1.38, 1.74) | 1.58 (1.34, 1.71) | 1.72 (1.64, 1.95) | <0.001 |
| Fibrinogen,g/L, median (IQR) | 3.42 (3.17, 3.47) | 3.38 (3.17, 3.44) | 3.42 (3.21, 3.47) | 0.091 |
| G, mmol/L, median (IQR) | 5.3 (4.7, 6.4) | 5.15 (4.68, 6.03) | 5.6 (4.95, 6.72) | 0.064 |
| TNF-α, median (IQR) | 9.77 (9.29, 10.66) | 9.66 (9.22, 9.83) | 15.89 (14.7, 16.32) | <0.001 |
| Hb, median (IQR) | 140 (131, 144) | 139 (131, 144) | 142 (131.5, 144) | 0.828 |
| Erythrocyte count, median (IQR) | 4.85 ± 0.51 | 4.86 ± 0.54 | 4.81 ± 0.45 | 0.591 |
| TG, mmol/L, median (IQR) | 1.37 (0.94, 2) | 1.43 (0.99, 2.22) | 1.37 (0.86, 1.83) | 0.171 |
| TC, mmol/L, median (IQR) | 4.41 (3.77, 5.26) | 4.32 (3.62, 5.47) | 4.53 (3.85, 5.16) | 0.744 |
| HDL, mmol/L, median (IQR) | 0.99 (0.81, 1.17) | 1.01 (0.84, 1.17) | 0.93 (0.8, 1.19) | 0.738 |
| LDL, mmol/L, median (IQR) | 2.59 ± 0.81 | 2.56 ± 0.8 | 2.67 ± 0.85 | 0.517 |
| APA, g/L, median (IQR) | 1.26 (1.06, 1.37) | 1.27 (1.06, 1.36) | 1.21 (1.06, 1.4) | 0.825 |
| APB, g/L, median (IQR) | 0.85 (0.67, 1.03) | 0.86 (0.68, 1.02) | 0.84 (0.67, 1.04) | 0.858 |
| **Hematoma location, n (%)** |  |  |  |  |
| Cerebellum, n (%) | 17 (13) | 13 (14) | 4 (10) | 0.750 |
| Basal ganglia, n (%) | 43 (33) | 33 (36) | 10 (26) | 0.349 |
| Intraventricular haemorrhage, n (%) | 11 (8) | 6 (7) | 5 (13) | 0.302 |
| Bleeding from other locations, n (%) | 53 (40) | 38 (41) | 15 (38) | 0.914 |
| Subarachnoid haemorrhage, n (%) | 19 (15) | 15 (16) | 4 (10) | 0.530 |
| Tracheotomy, n (%) | 41 (31) | 28 (30) | 13 (33) | 0.904 |
| Epidural tube, n (%) | 38 (29) | 23 (25) | 15 (38) | 0.18 |
| External ventricular drain, n (%) | 12 (9) | 6 (7) | 6 (15) | 0.181 |
| Intra-operative time, median (IQR) | 222.49 ± 43 | 222.15 ± 46.84 | 223.32 ± 32.73 | 0.87 |

**Abbreviations**: WBC, white blood cell; NLR, neutrophil to-lymphocyte ratio;G, glucose; PA, prealbumin; TC, triglyceride; TG, triglycerides; CRP, c-reactive protein; HDL, high-density lipoprotein; LDL, low-density lipoprotein; ApoA, apolipoprotein A; ApoB, apolipoprotein B; TNF-α, tumor necrosis factor-α
